# Two-electron oxygen reduction on fullerene C_60_-carbon nanotubes covalent hybrid as a metal-free electrocatalyst

**DOI:** 10.1038/s41598-019-50155-7

**Published:** 2019-09-24

**Authors:** Aliyeh Hasanzadeh, Alireza Khataee, Mahmoud Zarei, Yifeng Zhang

**Affiliations:** 1 Research Laboratory of Advanced Water and Wastewater Treatment Processes, Department of Applied Chemistry, Faculty of Chemistry, University of Tabriz, 51666-16471 Tabriz, Iran; 2 Health Promotion Research Center, Iran University of Medical Sciences, 1449614535 Tehran, Iran; 3 Research Laboratory of Environmental Remediation, Department of Applied Chemistry, Faculty of Chemistry, University of Tabriz, 51666-16471 Tabriz, Iran; 4 Department of Environmental Engineering, Building 115, Technical University of Denmark, DK-2800 Lyngby, Denmark

**Keywords:** Heterogeneous catalysis, Carbon nanotubes and fullerenes, Electrocatalysis

## Abstract

Nanocarbon materials are considered to be active for electrochemical oxygen reduction reaction (ORR) for hydrogen peroxide (H_2_O_2_) synthesis. In the present work, a new type of fullerene 60 (C_60_)-carbon nanotubes (CNTs) hybrid with covalently attached C_60_ onto outer surface of CNTs was synthesized. The structure of C_60_-CNT hybrid was confirmed by physical and chemical characterizations and its conformation is proposed featuring the covalent incorporation of CNTs and C_60_ derivative. C_60_-CNT hybrid showed high efficiencies on electro-generating H_2_O_2_, owing to huge surface area and intermolecular electron-transfer in the hybrid structure. A high H_2_O_2_ production rate of 4834.57 mg L^−1^ h^−1^ (426.58 mmol L^−1^) was achieved at − 0.2 V vs saturated calomel electrode (SCE).

## Introduction

Hydrogen peroxide (H_2_O_2_) is an eco-friendly and essential chemical that is widely used as an oxidizer, antiseptic and bleaching agent for a huge range of industrial processes^1^. The well-developed anthraquinone-based H_2_O_2_ production is the current method that has taken in multistep reactions and separations, energy-intensive and along with the production of organic wastes^2,3^. Furthermore, the use of noble‐metal based catalysts (Pt, Pd, Au, and Ag) and hydrogenation step under high-pressure H_2_ have a negative effect on the production costs^4^. These major disadvantages have triggered the interests in the development of more facile and green method for H_2_O_2_ generation. As a substitute route, H_2_O_2_ production through the reaction of O_2_ and H_2_ under a direct catalytic process has been proposed^5^. However, the use of toxic and expensive catalysts based on precious metals and the possible explosion of the O_2_ and H_2_ mixture made this approach unattractive for industrial applications^2,4^. By contrast, electrochemical H_2_O_2_ generation through the two-electron oxygen reduction reaction (ORR) is an appealing procedure that allows green, safe route, low-cost, and *in-situ* generation of H_2_O_2_ ^6–8^. However, H_2_O_2_ production from the ORR competes with the O_2_ reduction to H_2_O through a four-electron transfer, and thus, the main challenge lies in the development of efficient electrocatalysts that can selectively prefer the two-electron reduction pathway^1,9^.

Noble‐metal‐based catalysts with engineered reactive sites by means of various strategies, such as coating of their surfaces with amorphous carbon, supporting with single-atom catalysts, and alloying by inactive elements, have shown outstanding catalytic activity and selectivity for H_2_O_2_ generation^9,10^. Unlike these materials, metal‐free carbon materials have found remarkable research attention as low‐cost and conductive electrocatalysts. Furthermore, most of the carbon-based catalysts present a rather low overpotential for the two-electron oxygen reduction pathway^11^. In fact, dissociation of the potent O=O bond occurs in the four-electron pathway, whereas the O–O bond remains during the two-electron pathway^3^.

Applications of nanocarbon materials (e.g., graphene, carbon nanotubes (CNTs), and fullerenes) in numerous areas have received considerable attention due to their unique physicochemical properties^12^. Moreover, the incorporation of nanocarbons can develop the existing features or benefit from their excellent properties. Therefore, in recent years, efforts have been being made to develop various carbon nanomaterial hybrids with each other so as to extend their applications^13^. Nanocarbon hybrids such as graphene-CNT^14–17^ and C_60_-graphene^18^ are the promising electrocatalysts for the ORR because of their higher performance compared to that of their individual forms. The hybridizations of CNTs and C_60_ fullerene (or a fullerene derivative), which have been shown as the excellent electron acceptor, can be prepared through either non-covalent or covalent procedures^18–21^. However, covalent functionalization of C_60_ and its derivatives to the outer surface of the CNTs is more potent than the van der Waals interaction between them and thus can impose more remarkable changes on their band and electronic structures. Additionally, the covalent nature renders powerful intermolecular interactions between CNTs and C_60_ structures. A main challenge in this context is to develop the effective and convenient approaches for the synthesis of C_60_-CNT covalent hybrids. Here, a new type of C_60_-CNT covalent hybrid was prepared by Birch reduction^22^ reaction between multi-walled CNTs and 4-chlorobenzoic acid functionalized-fullerene (CB-C_60_) via Friedel-Crafts acylation.

Herein, the formation of a new C_60_-CNT hybrid based on the direct covalent linkage of C_60_ derivative molecules on the sidewalls of CNTs was explored, and the structural properties of the as-prepared hybrid were studied by means of microscopic and spectroscopic approaches. Subsequently, the selectivity and electrochemical activity of the C_60_-CNT hybrid were studied toward the two-electron ORR for H_2_O_2_ generation. Physical and chemical characterization tools were associated with obtained results from electrochemical analyses to clarify the distinctive features of as-prepared nanocarbon hybrid that contribute to the H_2_O_2_ electro-generation activity.

## Experimental Section

### Chemicals and materials

Hydrochloric acid (37%, Sigma-Aldrich®), lithium granular (98%, Sigma-Aldrich®), sulfuric acid (>95–97%, Merck, Germany), C_60_ (purity: >98%, Sigma-Aldrich®), 4-chlorobenzoic acid (CB) (>99%, Sigma-Aldrich®), multi-walled CNTs (~90% purity on carbon basis, size 8–15 nm outer diameter and 3–5 nm inner diameter, Cheap Tubes, USA) and phosphorous pentoxide (>98%, Merck, Germany) were used without further purification to prepare different solutions in Milli-Q water (resistivity ≥18.2 MΩ·cm at 25 °C).

### Functionalization of C_60_ with 4-chlorobenzoic acid

4-Chlorobenzoic acid (0.25 mmol), C_60_ (0.45 mmol), and polyphosphoric acid (PPA, 83% assay, 20 g) were added in a 250-mL resin flask containing a mechanical stirrer under nitrogen atmosphere. After being stirred at 130 °C for 3 h, 5 g of phosphorous pentoxide (P_2_O_5_) was added into the reaction media in one portion. The dark mixture turned into lighter brown. The resulting solution was further stirred at 130 °C for 48 h. Afterwards, the mixture was cooled down to reach the room temperature and it was slowly poured into deionized water to dissolve the PPA and P_2_O_5_. The precipitate was gathered by suction filtration and washed with NH_4_OH. Residual PPA and unreacted CB were eliminated by Soxhlet extraction with deionized water and methanol for four days, respectively. Finally, the sample was vacuum-dried at 60 °C for 12 h, as a result, the gray powder was obtained.

### Preparation of C_60_-CNT hybrid and mixture

**Figure 1:**
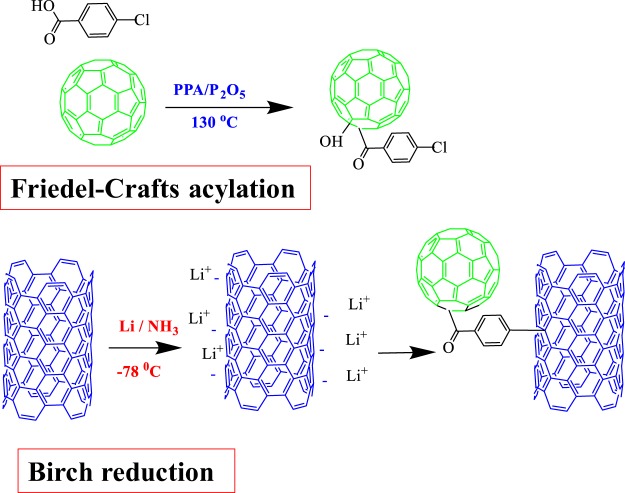
Synthesis procedure of CB-C_60_ and C_60_-CNT hybrid.

The preparation procedures using Birch reduction^22^ were as follows: 0.2 g of multi-walled CNTs were added into a dry 150-mL three-neck round-bottom vessel under argon atmosphere. Then, 60 mL of NH_3_ was condensed into the reaction medium, which was cooled down to −77 °C in a liquid nitrogen-butyl acetate bath. Subsequently, 1.2 g of lithium metal was gradually added to the reaction vessel while being stirred. 40 min after the lithium addition, 1.4 g of as-prepared CB-C_60_ sample was slowly added and the reaction mixture was endlessly stirred at -33 °C during 24 h. In order to quench the reaction, absolute ethanol was added followed by the addition of deionized water. The resulting suspension was acidified by adding 1.0 mol L^−1^ HCl and washed with absolute ethanol by centrifuging several times and finally dried at 80 °C for 8 h under vacuum (See Fig. 1).

### Fabrication of gas diffusion electrode

0.2 g of carbon nanomaterials (CB-C_60_, CNTs and C_60_-CNT hybrid), 0.42 g of PTFE, 3% V/V of n-butanol were thoroughly blended and dispersed in 30 mL of N-methyl-2-pyrrolidone (NMP) for 1 h to prepare the gas diffusion electrode (GDE) electrodes. The as-prepared mixture underwent heat treatment at 80 °C until a paste-like ink was obtained and pressed by a stainless steel mesh current collector at 10 MPa for 5 min. The prepared electrodes were sintered under N_2_ atmosphere at 350 °C for 30 min, followed by cutting to diameter of 15 mm. The obtained electrodes were put at the bottom of a polypropylene cylindrical holder for manufacturing the GDEs. A graphite disk, which is in contact with a copper wire as the electrical connector, is located in the cylindrical holder.

### Physical and chemical characterization methods

TESCAN (Mira3, Czech Republic) and JEOL (JEM-2200FS, Japan) microscopes were used to record the scanning electron microscopy (SEM) and high resolution transmission electron microscopy (HR-TEM) micrographs, respectively. X-ray diffraction (XRD) analysis was done by Siemens D5000 X-ray diffractometer (Germany) using Cu Kα exciting source (λ = 1.54056 Å). Raman spectra were acquired by a WiTech confocal Raman microscope equipped with a 532 nm NiYAG laser. A Tensor 27 IR-spectrometer (Bruker, Germany) on the KBr pellets over the range of 400–4000 cm^−1^ was utilized for taking the Fourier transform infrared spectroscopy (FT-IR) spectra. Brunauer, Emmett and Teller (BET) method was performed using nitrogen adsorption/desorption at 77 K with a Belsorp mini II device (Bel, Japan). Atomic force microscopy (AFM) was applied to study the surface topography of the coated electrodes, which was performed by a Nanosurf Mobile S microscope (Nanosurf, Switzerland). The elemental composition was studied X-ray photoelectron spectroscopy (XPS) (PHI 5000 Versaprobe, Al Kα source). The water contact angles of the prepared electrodes were determined using a standard goniometer (200, Rame-Hart, USA). For this aim, 5 μL water droplet were deposited onto surface of prepared electrodes at ambient temperature. For each sample, at least five contact angle measurements were averaged on different areas of electrode surface.

### Electrochemical characterization

The electrochemical measurements were done by means of a computer-controlled potentiostat (PARSTAT 2273). For the rotating ring disk electrode (RRDE) measurements, a three-electrode system was assembled with a standard three-electrode configuration. The as-prepared catalyst ink, consisting of nanocatalyst powder, water, isopropanol and Nafion solution (5 wt%), was carefully dropped on the RRDE electrode (the electrode area is 0.2475 cm^2^) with a catalyst loading of 0.1 mg cm^−2^ as working electrode. Graphite rod and saturated calomel electrode (SCE) were used as counter electrode and reference electrode, respectively. For the accurate and reproducible measurement of H_2_O_2_ selectivity, it is very important to clean the RRDE thoroughly prior to each experiment. Cyclic voltammetry (CV) was performed between −1.2 and 0.3 V (vs. SCE) in N_2_-saturated 0.5 mol L^−1^ H_2_SO_4_ at a scan rate of 5–10 mV s^−1^, in which a steady CV response was obtained. O_2_ gas was purged into the electrolyte for 5 min (caution: if the time interval between the Pt ring cleaning and ORR measurement is long, the H_2_O_2_ selectivity can be underestimated due to the surface passivation of the Pt ring). The electrochemical impedance spectroscopy (EIS) was conducted at −0.2 V (vs. SCE) from 100,000 to 1 Hz to determine the uncompensated resistance (R_u_) in a high-frequency range for iR-correction. The H_2_O_2_ production activity was assessed by linear sweep voltammetry (LSV) in O_2_-saturated 0.1 mol L^−1^ H_2_SO_4_ at a scan rate of 5 mV s^−1^ and a rotation speed of 1600 rpm. The ring electrode was set at a constant potential of 0.5 V vs. SCE to detect the generated H_2_O_2_. The electron transfer numbers (*n*) and H_2_O_2_ selectivity were calculated using the following relations^23^:

1 $$n=\frac{4{{\rm{NI}}}_{{\rm{d}}}}{{{\rm{NI}}}_{{\rm{d}}}+{{\rm{I}}}_{{\rm{r}}}}$$

2 $${{\rm{H}}}_{2}{{\rm{O}}}_{2}\, \% =\frac{200{{\rm{I}}}_{{\rm{r}}}}{{{\rm{NI}}}_{{\rm{d}}}+{{\rm{I}}}_{{\rm{r}}}}$$

where I_r_ and I_d_ denote the ring current and disk current, respectively. The N was the collection efficiency of Pt ring, which was determined to be 0.3 with [Fe(CN)_6_]^4−/3−^ redox probe^24^.

The Koutecky-Levich (K-L) plots (J^−1^ versus ω^−1/2^) represents the relation between the measured current, electron transfer number, and rotation speed as follows:

3 $$\frac{1}{{\rm{J}}}=\frac{1}{{{\rm{J}}}_{{\rm{K}}}}+\frac{1}{{B{\rm{\omega }}}^{1/2}}$$

4 $${\rm{B}}=0.62\,{\mathrm{nF}{\rm{\nu }}}^{-1/6}{{\rm{C}}}_{{{\rm{O}}}_{2}}{{\rm{D}}}_{{{\rm{O}}}_{2}}^{3/2}$$

where J, J_K_, ω, n, F, ʋ, ${{\rm{C}}}_{{{\rm{O}}}_{2}}$, and ${{\rm{D}}}_{{{\rm{O}}}_{2}}$ indicate the determined current, kinetic current densities (mA cm^−2^), rotation rate (rad s^−1^), number of transferred electrons for ORR, Faraday constant (96485.34 C mol^−1^), viscosity of electrolyte (0.01 cm^2^/s), oxygen concentration in the electrolyte (1.26 × 10^−6^ mol cm^−3^), and oxygen diffusion coefficient in electrolyte (1.93 × 10^−5^ cm^2^ s).

The stability of as-prepared C_60_-CNT hybrid was evaluated using the chronoamperometric method performed at a constant potential of −0.2 V vs. SCE. The H_2_O_2_ faradaic efficiency was determined from the H_2_O_2_ yield against the quantity of charge passed:

5 $${{\rm{H}}}_{2}{{\rm{O}}}_{2}\,{\rm{faradaic}}\,{\rm{efficiency}}\,( \% )=2{\rm{CVF}}/{\rm{Q}}$$

where C is the H_2_O_2_ concentration (mol L^−1^), V is the volume of electrolyte (L), F is the Faraday constant (96485.3 C mol^−1^), and Q is the passed charge amount (C).

To appraise the number of electrochemically active centers on the surface of as-prepared hybrid electrodes, CV analysis in a solution containing potassium hexacyanoferrate-III (1 mmol L^−1^) and potassium chloride (1 mol L^−1^) was fulfilled. The Randles-Sevcik equation^25^ (Eq. 6) was applied to calculate electrochemically active surface area (ECSA) of the mentioned electrodes.

6 $${{\rm{I}}}_{{\rm{P}}}=2.65\times {10}^{5}{{\rm{n}}}^{3/2}{{\rm{ACD}}}^{1/2}{{\rm{\upsilon }}}^{1/2}$$

where I_p_ is the peak current (A), n (=1) is the number of electrons transferred, A is the effective area of the electrode(cm^2^), D is the diffusion coefficient of potassium hexacyanoferrate-III (taken to be 7.60 × 10^−6^ cm^2^ s^−1^ at 25 °C)^26^, C is the concentration (mol cm^−3^), υ is the scan rate (V s^−1^).

Nafion 117 membrane was applied as a separator in H_2_O_2_ electro-generation experiments which were carried out in a cell with two portions. The cathodes were chosen in the form of pure carbon paper, improved cathode with CB-C_60_, CNTs and C_60_-CNT hybrid electrodes (with similar area of 4.9 cm^2^) and anode was selected in the form of Pt sheet (10 cm^2^). For electro-generation of H_2_O_2_, the diffusion cathode was steadily provided with pure O_2_ gas. 100 mL of Na_2_SO_4_ solution with specific concentrations was magnetically stirred (at 300 rpm) and produced as a supporting electrolyte for all tests. The 3100ST pH meter was used to identify the solution pH (Ohaus, Switzerland). The pH was set by H_2_SO_4_ and NaOH solutions (0.1 mol L^−1^). The concentration of electro-generated H_2_O_2_ was acquired by spectrophotometer based on iodide approach^27^. In this method, the sample (4 mL), potassium hydrogen phthalate (3 mL, 0.5 mol L^−1^) and iodide reagent (3 mL) which contains 0.4 mol L^−1^ KI, 10^−4^ mol L^−1^ (NH_4_)_2_MoO_4_ and 0.05 mol L^−1^ NaOH were mixed. Afterwards, the solution absorbance was read at 351 nm by the UV–Vis spectrophotometer (DR3900, Hach, USA). H_2_O_2_ quantity was obtained by flow-injection chemiluminescence method following the luminol reaction by H_2_O_2_.

## Results and Discussion

### Functionalization and characterization of CB-C_60_

C_60_ was functionalized with CB through direct Friedel-Crafts acylation method in a PPA/P_2_O_5_ medium (Fig. 1). ^1^H, ^13^C NMR, and FT-IR spectroscopic techniques were applied to monitor the functionalization progress. To remove possible impurities, samples were worked-up by adding water and methanol in the Soxhlet extraction for four days to eliminate any residual precursor before characterization. The ^1^H NMR spectrum of CB-C_60_ in CS_2_/CDCl_3_ (Fig. S1a) indicated four peaks with chemical shifts centered at 5.28 (s, 1H), 7.87 (d, *J* = 8.4 Hz, 2H), 8.25 (d, *J* = 8.4 Hz, 2H). A single peak placed at 𝛿 = 5.28 ppm, consistent with hydroxyl proton (C-OH), was further validated by deuterium (H/D) exchange in which the peak vanished by addition of D_2_O and a new peak of water protons appeared at 𝛿 = 4.78 ppm (Fig. S1b). The ^13^C NMR spectrum (Fig. S1c) demonstrates two peaks at 64.31 (C_60_-O) and 82.38 ppm (C_60_-C) for the sp^3^ carbons, 30 signals including some overlapping ones over the range of 125 to 151 ppm for the carbons of C_60_ structure (sp^2^-C) and a singlet peak at 196.65 ppm corresponding to ketone group (C=O).

For further investigation of functionalization of C_60_ with CB, FT-IR spectra of C_60_, CB, and CB-C_60_ were taken (Fig. S1d). The bare C_60_ spectrum exhibited four main dominant peaks at 525, 574, 1180, and 1428 cm^−1^, which could be related to the F_1u_ infrared active vibrations^28,29^. CB spectrum showed a strong peak at 1690, a weaker peak at 1230 cm^−1^ and a broad peak at about 3200–3600 cm^−1^, which were attributed to carboxylic acid group (C=O, C–OH, O–H stretching modes, respectively). In the spectrum of the CB-C_60_, the appearance of a peak at 1718 cm^−1^ was ascribed to the stretching vibrations of carbonyl group (C=O), which demonstrated that the attachment between CB and C_60_ molecules had indeed been attained by acylation. In addition, two peaks were detected in the spectrum of the CB-C_60_ at 545 and 582 cm^−1^, which are assumed to be resulting from the peaks of C_60_ at 525 and 572 cm^−1^, respectively. Furthermore, the peaks observed around 755 cm^−1^ and in the range of 1400–1600 cm^−1^ was assigned to the vibration of C–Cl and C=C aromatic, respectively. These obtained results verify that C_60_ has been covalently functionalized by CB as a hydroxyfullerenyl ketone.

**Figure 2:**
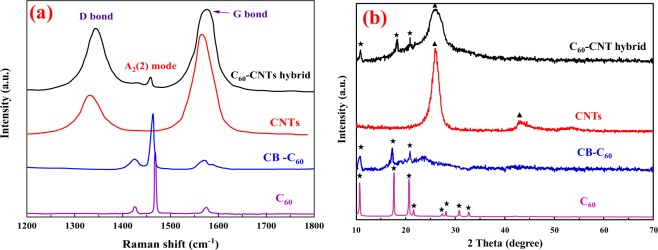
(**a**) Raman and (**b**) XRD spectra recorded for C_60_, CB-C_60_, CNTs, and C_60_-CNT hybrid.

Raman spectroscopy analysis is commonly applied to study the lattice and electronic structures of nanocarbon materials. Typical Raman spectra of raw C_60_ and as-synthesized CB-C_60_ are indicated in Fig. 2a. The spectrum of the raw C_60_ exhibited the A_g_(2) pentagonal pinch vibration frequency at 1469 cm^−1^, and the additional H_g_(7) and H_g_(8) modes can be distinguished at 1425 and 1575 cm^−1^, respectively^30^. In the spectrum of CB-C_60_, because of the covalent bonding of the CB to the C_60_ cage the positions of relevant modes were red-shifted by 6 cm^−1^ with respect to that in the bare C_60_. Moreover, H_g_(7) and H_g_(8) modes of CB-C_60_ appeared notably broadened, owing to the decreased symmetry of CB-C_60_ compared to the pristine C_60_. It can be seen that the A_g_(2) mode of CB-C_60_ spectrum had a slight broadening, which can be ascribed to inhomogeneous broadening phenomenon as it is a non-degenerate mode and hence cannot split upon modification of the C_60_ cage^31^.

### Characterization of C_60_-CNT hybrid

A series of spectroscopic and morphological surveys demonstrated the as-prepared C_60_-CNT hybrid structure. Raman spectroscopy was used to verify the covalent incorporation of C_60_ to sidewall of CNTs. As can be realized from Fig. 2a, the Raman spectrum of pristine CNTs showed two peaks at 1347 and 1578 cm^−1^ as D-band and G-band, respectively. The G-band was associated to the sp^2^ hybridization of carbon in the graphitic frame. The D-band mainly originates from the vibrations of sp^3^ bonds of carbon atoms which show the disorders and defects in the CNTs^32^. In the spectrum of the C_60_-CNT hybrid, besides the D and G bands at 1350 and 1582 cm^−1^, respectively, a peak located at 1458 cm^−1^ was clearly detected, which can be ascribed to the A_g_(2) mode of the C_60_ framework^33^. However, the up-shift of the D and G peak positions and down-shift of the A_g_(2) mode in the C_60_-CNT hybrid spectrum compared to the individual materials showed that the charge-transfer from CNTs to C_60_ may happen as a result of strong electron-withdrawing ability of C_60_ ^34^, which was in agreement with the reported results^19,20^. On the other hand, the intensity ratio of these bands (I_D_/I_G_) was proportional to the disorder degree on the carbon matrix and applied as a probe to identify the covalent attachment of the CNTs surface. The I_D_/I_G_ value of the C_60_-CNT hybrid (0.96) was greater than that of the CNTs (0.43), indicating that the CB-C_60_ covalently bonded onto the CNTs surface^33^. These results readily concur with those obtained from FT-IR analysis (see ESI Fig. S2).

XRD diffractograms of C_60_, CB-C_60_, CNTs and C_60_-CNT hybrid are revealed in Fig. 2b. For bare C_60_, the localized peaks at 2θ = 10.8°, 17.7°, 20.8°, 21.7°, 27.5°, 28.2°, 30.8° and 32.7° that referred to plane reflections of (111), (220), (311), (222), (331), (420), (422) and (511), respectively, associated with face centered cubic (fcc) crystalline phase of C_60_ with lattice constant a = 14.17 Angstrom (JCPDS 44-0558)^35,36^. As for CB-C_60_, the detected peaks at 2θ = 10.2°, 17.1°, 20.2°, and 21.5° became slightly broader with decreased intensity compared to those of the unfunctionalized C_60_, indicating that functionalization of C_60_ did not completely change the lattice structure of C_60_. As can be observed in Fig. 2b, the pure CNTs^37^ displayed two broad peaks at 2θ = 25.74° and 42.87° which can be ascribed to the hexagonal graphite crystal planes (002) and (001), respectively, with an interlayer distance (d) of 0.34 nm. XRD diffractogram of C_60_-CNT hybrid demonstrates a superposition of the peaks of the CB-C_60_ and CNTs, evidencing the hybrid structure of these two nanocarbons. The intensity of the (002) diffraction peak at 26.12° remarkably decreased in comparison to the pure CNTs with negligible shift. However, the weaker peak at 42.87° (001) of CNTs pattern almost disappeared in the C_60_-CNT hybrid illustrating that the lattice structure of CNTs was relatively changed upon the linkage of the CB-C_60_ moiety. On the other hand, in comparison to the XRD analysis of CB-C_60_, the diffraction peaks of C_60_-CNT hybrid centered at 10.5°, 18.1° and 20.9° appeared which asserted the formation of C_60_-CNT hybrid. For further investigation, the XRD pattern for the mixture of CNTs and bare C_60_ (C_60_-CNT mixture) was taken, which indicated a poorly crystalline or amorphous feature to that of CNTs and peaks of C_60_ that were hardly detected (see ESI Fig. S3). It was confirmed that physical mixing of C_60_ with CNTs slightly influenced the properties of CNTs compared to covalent hybrid structure. In fact, in the mixture, C_60_ molecules were attached to the CNTs surface by van der Waals forces^38,39^.

**Figure 3:**
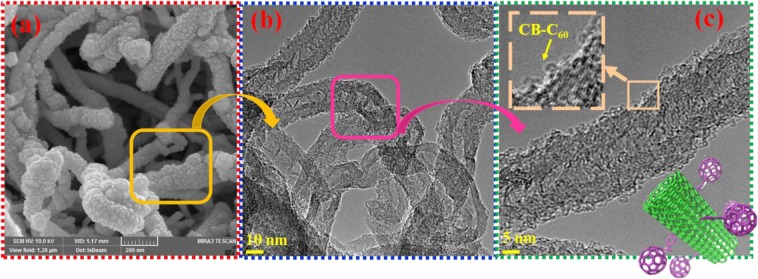
(**a**) SEM and (**b,c**) TEM images of C_60_-CNT hybrid.

The SEM micrographs of synthesized C_60_-CNT hybrid (Fig. 3a) demonstrate the growth in wall width of C_60_-CNT hybrid in comparison with unmodified CNTs (SEM and TEM images are shown in Fig. S4) which can be attributed to the addition CB-C_60_ on the CNTs surface. In addition, as observed by the HR-TEM image of C_60_-CNT hybrid in Fig. 3b,c, the spherical CB-C_60_ molecules are covalently conjugated on the CNTs sidewall. The diameter of the individual CB-C_60_ sphere was determined to be around 1 nm (Fig. 3c, inset), while TEM micrographs of spherical particles of C_60_ (Fig. S5a) and CB-C_60_ (Fig. S5b) display the facile agglomeration of their molecules resulted from the potent van der Waals attractions and profound π-π forces between them^40^. It should be noted that for removing the unreacted and physically adsorbed CB-C_60_ particles, the synthesized samples were washed via ultrasonication in CS_2_ solvent several times.

**Figure 4:**
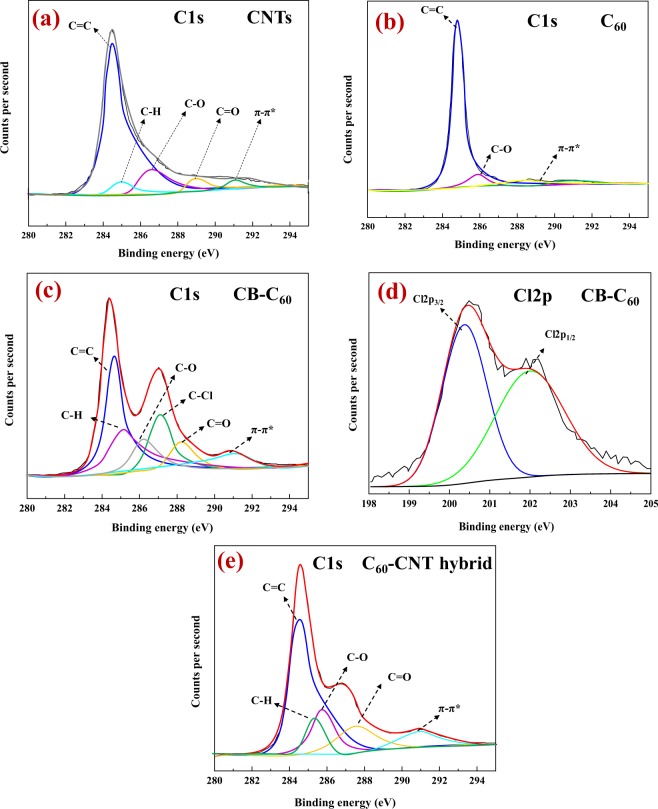
Deconvoluted XPS plots: C 1s core level of (**a**) pristine CNTs; (**b**) C_60_; (**c**) CB-C_60_; (**d**) Cl2p region of the structure in c; and (**e**) C 1s core level of C_60_-CNT hybrid.

XPS analysis was fulfilled to clarify the bonding configurations and the chemical composition in synthesized C_60_-CNT hybrid. The C 1s scan of the pristine CNTs (Fig. 4a) indicated the C–C bonds (sp^2^ carbon) at 284.4 eV, together with different peaks located at 284.9, 286.1, and 288.0 eV could be connected to the C–H, C–O, and C=O bonds, respectively, because of native surface groups and structural defects present in the raw CNTs sample. A minor peak at 290.9 eV can be ascribed to the π–π* changes in sp^2^ carbon structures^18^. The XPS spectrum of C1s of C_60_ shows the located peaks at 284.6 eV (C–C), 286.1 eV (C–O), and two π–π* shake-up peaks around 290 eV (Fig. 4b). As it can be observed in Cls region of CB-C_60_ (Fig. 4c), three new peaks appear at 287.1, 285.3, and 288.0 assigned to aromatic C–Cl, C–OH, and C=O bonds, respectively. XPS spectrum of Cl2p (Fig. 4d) indicates two major peaks centered at 200.5 eV (Cl2p_1/2_) and 202.1 eV (Cl2p_3/2_). The results can confirm the successful functionalization of C_60_ within the CB. In case of C_60_-CNT hybrid (Fig. 3e), the peaks at 288.0 and 285.3 eV assigned to C=O and C–OH groups, respectively^18^, indicating a higher relative intensity than those of the pristine CB-C_60_ and CNTs. It is suggesting that more C=O and C–OH bonds have been introduced to the hybrid structure. These results readily concur with those obtained from the characterizations discussed above.

### Physical characterization of fabricated GDE cathodes

**Figure 5:**
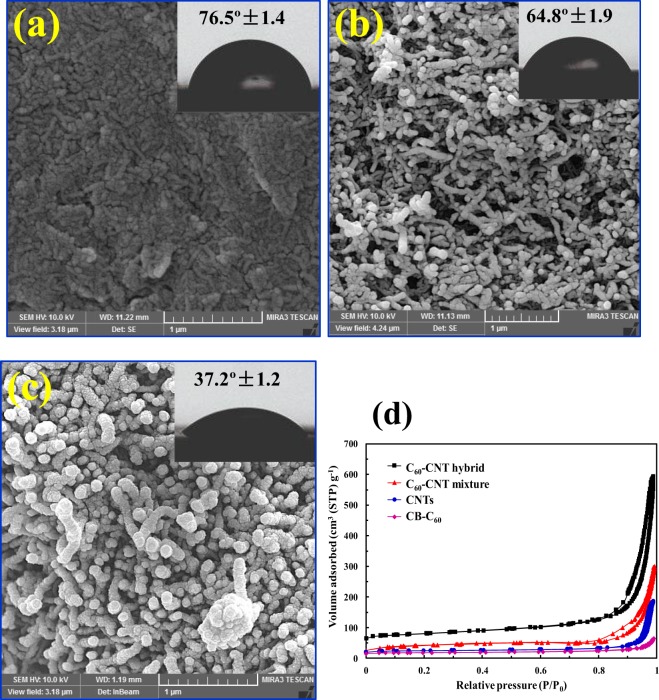
SEM images of the fabricated electrodes with insetting of water contact angle images (**a**) CB-C_60_; (**b**) CNTs; (**c**) C_60_-CNT hybrid, and (**d**) N_2_ adsorption/desorption isotherms of CB-C_60_, CNTs, C_60_-CNTs mixture, and C_60_-CNT hybrid.

SEM micrographs of prepared electrodes by CB-C_60_ (Fig. 5a), CNTs (Fig. 5b), and C_60_-CNT hybrid (Fig. 5c) indicate the mesoporous morphology of CNTs and the slightly smooth plate surface of CB-C_60_, while C_60_-CNT hybrid electrode possesses a rough surface and heterogeneous porous structure. These results were further confirmed by means of BET analysis (Fig. 5d), wherein C_60_-CNT hybrid presented a higher surface area (422.52 m^2^ g^−1^) compared to that of the CNTs (185.28 m^2^ g^−1^), C_60_-CNT mixture (258.5 m^2^ g^−1^), and CB-C_60_ (98.2 m^2^ g^−1^), respectively.

The hydrophilic feature of the fabricated nanocarbon based electrodes was investigated by water contact angle (Fig. 5a–c, inset). The average contact angles of CB-C_60_, CNTs, and C_60_-CNT hybrid were found to be 76.5°, 64.8°, and 37.2°, respectively. As can be seen, the decrease in contact angle value means the increase hydrophilicity of the samples, which could be caused by the presence of carboxyl and hydroxyl groups in the fabricated C_60_-CNT hybrid electrode, as verified in the Raman and XPS data.

**Figure 6:**
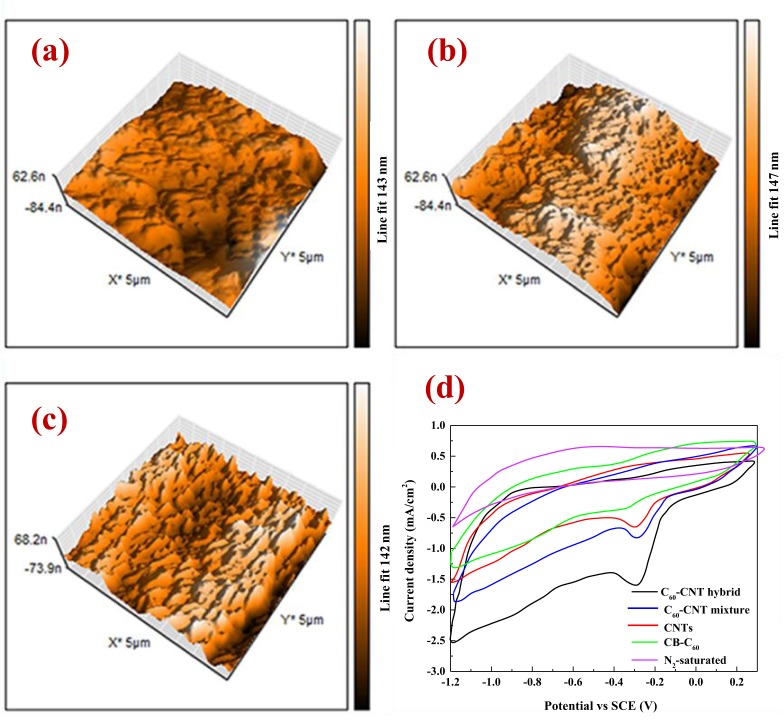
3D AFM images of the fabricated electrodes with (**a**) CB-C_60_; (**b**) CNTs; (**c**) C_60_-CNT hybrid, and (**d**) Cyclic voltammograms of CB-C_60_, CNTs, C_60_-CNT mixture, C_60_-CNT hybrid electrodes in O_2_ saturated solutions and C_60_-CNT hybrid in N_2_ saturated solution. Experimental conditions for CV: room temperature, [Na_2_SO_4_] = 0.05 mol L^−1^, pH = 4, and scan rate = 10 mV s^−1^.

The surface morphology of the CB-C_60_ (Fig. 6a), CNTs (Fig. 6b), and C_60_-CNT hybrid (Fig. 6c) electrodes were examined using AFM analysis, and the 3D images in the scale of 8 × 8 µm^2^ are displayed. In these graphs, dark areas show the pores or valleys, while bright areas show the highest point of the fabricated electrodes surface. The parameters of roughness were measured by the AFM images. The observed increase in the average roughness of the fabricated electrode with C_60_-CNT hybrid could be attributed to the increased surface porosity as shown in the SEM images and BET analysis.

### Electrochemical activity of oxygen reduction

In the first step, the electrochemical activities of the fabricated hybrid as the GDE materials towards ORR were evaluated by CV method in N_2_- or O_2_-saturated acidic media. As can be seen from Fig. 6d, there were no obvious reduction peaks in the N_2_-saturated solution using C_60_-CNT hybrid electrode, while there were well-known oxygen reduction peaks for all nanocarbon based materials in the O_2_-saturated solution, suggesting their distinct electrocatalytic activity toward ORR. Furthermore, the synthesized C_60_-CNT hybrid demonstrated the highest reduction current density of 2.2 mA cm^−2^, followed by CB-C_60_ (0.56 mA cm^−2^), CNTs (0.85 mA cm^−2^), and C_60_-CNT mixture (1.2 mA cm^−2^).

**Figure 7:**
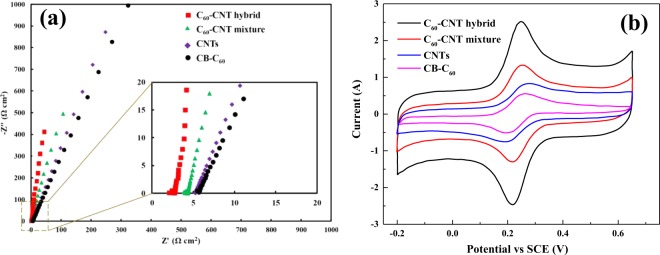
(**a**) EIS data with a frequency range between 100 kHz and 0.1 Hz, and (**b**) Cyclic voltammograms of in 1 mmol L^−1^ [Fe(CN)_6_]^3−/4−^ and 1 mol L^−1^ KCl solution at scan rate of 10 mV s^−1^, for CB-C_60_, CNTs, C_60_-CNTs mixture, and C_60_-CNT hybrid electrodes.

The onset potential for CB-C_60_, CNTs, C_60_-CNT mixture, and C_60_-CNT hybrid was −0.32, −0.28, −0.21 and −0.11 (V vs. SCE), respectively. These results suggest that the fabricated C_60_-CNT hybrid cathode had the best ORR activity amongst all the as-prepared nanocarbon catalysts. Besides, the EIS of all the samples was carried out in O_2_-saturated 0.5 mol L^−1^ Na_2_SO_4_ electrolyte solution (Fig. 7a). Evidently, the C_60_-CNT hybrid indicated a lower resistance foe mass- and charge-transfer than those of CB-C_60_, CNTs, C_60_-CNT mixture samples, showing a more promising reactant diffusion and electron transfer for ORR. These observations confirmed that the covalent integration of CB-C_60_ molecules into CNTs structure could reduce the resistance of charge transfer because of charge-transfer from CNTs to CB-C_60_, which was effective for improving catalytic activity toward ORR.

To appraise the number of electrochemically active centers on the surface of the fabricated nanocarbon based electrodes, CV analysis in a solution containing potassium ferrocyanide was performed^25^. The obtained voltammograms (Fig. 7b) demonstrate the high electrochemical performance of the C_60_-CNT hybrid electrode compared to others, where the electrochemical active surface area of C_60_-CNT hybrid was 27.78 ± 0.45 cm^2^ (Fig. S6).

#### Selectivity of hydrogen peroxide generation

**Figure 8:**
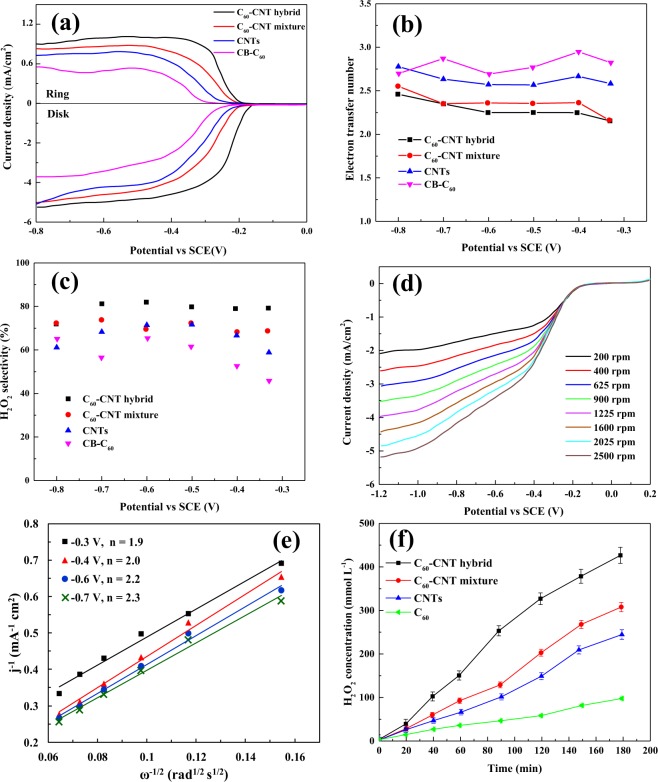
(**a**) RRDE polarization curves at 1600 rpm in O_2_-saturated 0.05 mol L^−1^ H_2_SO_4_ solution with a scan rate of 10 mV s^−1^; (**b**) H_2_O_2_ selectivity; (**c**) electron transfer number of CB-C_60_, CNTs, C_60_-CNT mixture, and C_60_-CNT hybrid, and (d) RDE of C_60_-CNT hybrid at O_2_-saturated Na_2_SO_4_ solution at a scan rate of 50 mV s^−1^, pH = 3; (**e**) K-L plots at different potentials and (**f**) H_2_O_2_ electrogeneration on the CB-C_60_, CNTs, C_60_-CNT mixture, and C_60_-CNT hybrid electrodes (pH = 3, [Na_2_SO_4_] = 0.05 mol L^−1^, and V = −0.2 V vs SCE).

The selectivity of the fabricated nanocarbon electrocatalysts toward ORR to H_2_O_2_ was evaluated by the RRDE method, using LSV at 1600 rpm rotating speed. As shown in Fig. 8a, among the material, C_60_-CNT hybrid presented the highest overall electocatalytic activity for ORR with disk current density (j_disk_) about −5.3 mA cm^−2^, and the main hydroperoxyl productivity under ring current density (j_ring_) around 0.91 mA cm^−2^ at −0.4 V (vs. SCE). Furthermore, C_60_-CNT hybrid exhibited the most positive onset potential around −0.12 V (vs. SCE). Figure 8b,c indicate the number of electrons transferred and the H_2_O_2_ selectivity trends resulting from the RRDE voltammograms, respectively. In the potential range of −0.3 to −0.8 V (vs. SCE), the mean number of transferred electrons was determined to be close to two (Fig. 8b), suggesting that ORR predominated by two-electron reduction pathway. The H_2_O_2_ selectivity within the studied applied potential range followed the order of C_60_-CNT hybrid > C_60_-CNT mixture > CNTs > CB-C_60_.

RDE analyses were further used to prove the ORR kinetics of C_60_-CNT hybrid. As shown in Fig. 8d, higher current intensities were obtained by increasing the rotation rate for the reduction of oxygen due to the decrease in the diffusion layer^23^. The K-L plot (Fig. 8e) with a good linearity at different potentials proved first-order reaction kinetics of the ORR process. In case of the fabricated C_60_-CNT hybrid, the mean number of transferred electrons per O_2_ molecule was measured to be ~2.2 (−0.3 to −0.7 V) based on the obtained slope of K-L plots.

For further evaluation of electrocatalytic performance, H_2_O_2_ generation rate of the fabricated nanocarbon based electrodes was investigated in O_2_-saturated acidic solution under the obtained optimal conditions (pH = 3 at −0.2 V vs SCE). As showed in Fig. 8f, the H_2_O_2_ concentration increased versus reaction times for all as-prepared nanocarbons. Notably, the C_60_-CNT hybrid electrocatalysts produced 426.58 mmol L^−1^ of H_2_O_2_ within 3 h and displayed a remarkably high H_2_O_2_ electrogeneration rate of 4834.57 mg L^−1^ h^−1^. This amount was greater than those of C_60_-CNT mixture (307.79 mmol L^−1^, 3488.28 mg L^−1^ h^−1^), CNTs (244.60 mmol L^−1^, 2772.13 mg L^−1^ h^−1^) and CB-C_60_ (97.72 mmol L^−1^, 1107.49 mg L^−1^ h^−1^).

It is noticeable that the faradaic efficiency for H_2_O_2_ production on C_60_-CNT hybrid electrode could reach 82.6% in the investigated applied potential, which is greater than those of previous studies of electrocatalysts under the same experimental conditions (Table S1). The observations show that the C_60_-CNT hybrid was a promising cathode material for H_2_O_2_ electrogeneration.

**Figure 9:**
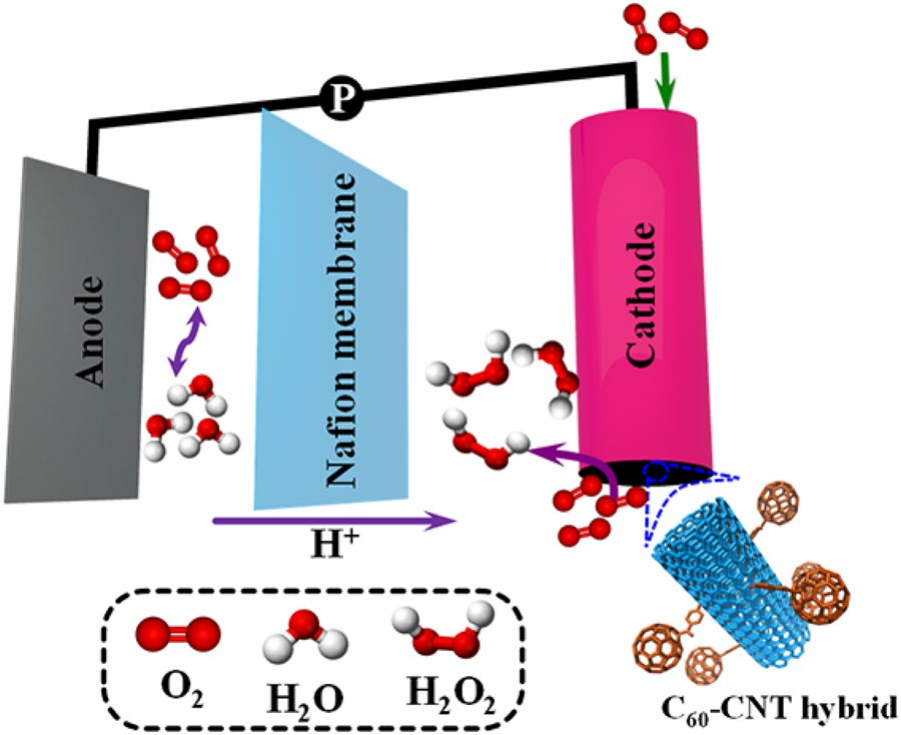
Schematic illustration of the H_2_O_2_ production by the C_60_-CNT hybrid electrode.

The higher ORR activity of the C_60_-CNT hybrid can be ascribed to the following reasons: (a) The carbon nanostructured morphology could provide higher active centers on the surface of electrode; (b) CB-C_60_ structures, as the electron acceptor, facilitate the electron transportation inside the hybrid and (c) the covalent attachment of CB-C_60_ into CNTs framework facilitates the oxygen adsorption and OOH desorption pathways^18^. The schematic of H_2_O_2_ production by C_60_-CNT hybrid electrode is represented in Fig. 9.

**Figure 10:**
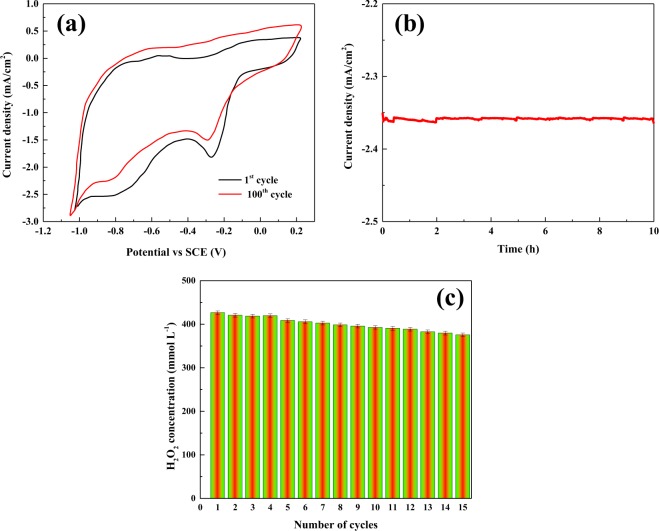
(**a**) Cyclic voltammetry of C_60_-CNT hybrid electrode for various cycles ([Na_2_SO_4_] = 0.05 mol L^−1^, pH = 3, and scan rate = 10 mV s^−1^); (**b**) chronoamperometric (current-time) response of C_60_-CNT hybrid in O_2_ saturated; and (**c**) H_2_O_2_ electrogeneration on C_60_-CNTs hybrid electrode for 15 repeated runs; [Na_2_SO_4_] = 0.05 mol L^−1^, pH = 3 and V = −0.2 V (vs SCE).

The stability of as-prepared electrodes is one of the most noteworthy parameters to evaluate their performance toward two-electron ORR. The stability of C_60_-CNT hybrid electrode was studied by sequential scanning of potential for 100 cycles (Fig. 10a), the chronoamperometric method for 10 h (Fig. 10b), and H_2_O_2_ generation rate over 15 repeated runs (Fig. 10c). As can be observed, C_60_-CNT hybrid even shows well two-electron ORR efficiency in the 100^th^ cycle compared to the 1^st^ cycle. Moreover, the peak current in the chronoamperometric curve, after a prolonged operation, about 98% of the initial current is retained and the rate of H_2_O_2_ generation after 15 runs (382.85 mmol L^−1^) was close to 426.58 mmol L^−1^. These obtained results proved high stability of the C_60_-CNT hybrid electrode for two-electron ORR.

## Conclusion

In summary, we demonstrated a method to synthesize the covalent C_60_-CNT hybrid as a novel electrocatalyst for H_2_O_2_ production. The C_60_-CNT hybrid exhibited high content of large surface area, intermolecular electron transitions, fast mass transport, and defect sp^3^-C bonds. It was demonstrated here the high performance of C_60_-CNT hybrid, as a cathode electrode, for electrogeneration of H_2_O_2_ (112.6–792.6 mmol h^−1^ g^−1^). In addition, the C_60_-CNT hybrid showed high stability and reusability in ORR. This study may provide a new insight into the design of metal-free and efficient nanocarbon-based electrocatalysts for production of H_2_O_2_.

## Supplementary information

Supporting Information
