# Supplementary material for: Two-electron oxygen reduction on fullerene C60-carbon nanotubes covalent hybrid as a metal-free electrocatalyst
Source: Sci Rep. 2019 Sep 24;9:13780. doi: 10.1038/s41598-019-50155-7 (PMC6760168; doi:10.1038/s41598-019-50155-7)
Supplement: Supplementary file 1 — Supporting Information [file 41598_2019_50155_MOESM1_ESM.docx]

**Supporting Information**

**Two-electron oxygen reduction on fullerene C_60_-carbon nanotubes covalent hybrid as a metal-free electrocatalyst**

Aliyeh Hasanzadeh **^a^**, Alireza Khataee **^a,b,*^**, [Mahmoud Zarei](https://www.sciencedirect.com/science/article/pii/S0045653518302637#!) **^c^**, Yifeng Zhang **^d^**

*^a^ Research Laboratory of Advanced Water and Wastewater Treatment Processes, Department of Applied Chemistry, Faculty of Chemistry, University of Tabriz, 51666-16471 Tabriz, Iran*

*^b^ Health Promotion Research Center, Iran University of Medical Sciences, 1449614535 Tehran, Iran*

*^c^ Research Laboratory of Environmental Remediation, Department of Applied Chemistry, Faculty of Chemistry, University of Tabriz, 51666-16471, Tabriz, Iran*

*^d^ Department of Environmental Engineering, Building 113, Technical University of Denmark, DK-2800 Lyngby, Denmark*

^*^Corresponding author:

Tel.: +98 41 33393165; Fax: +98 41 33340191.

E–mail address: a_khataee@tabrizu.ac.ir

**Calculation formula for H_2_O_2_ production rate:**

The production rate of H_2_O_2_ were calculated using the following equations: $H_{2}O_{2} production rate (mg L^{-1}h^{-1}) =C_{H_{2}O_{2}}\times\frac{M_{w}}{t}$ (S1)

where $C_{H_{2}O_{2}}$ is the generated concentration of H_2_O_2_ (mmol L^-1^), M_w_ is the molecular weight of H_2_O_2_ (M = 34.01 g mol^−1^), and t is the electrochemical reaction time (h).


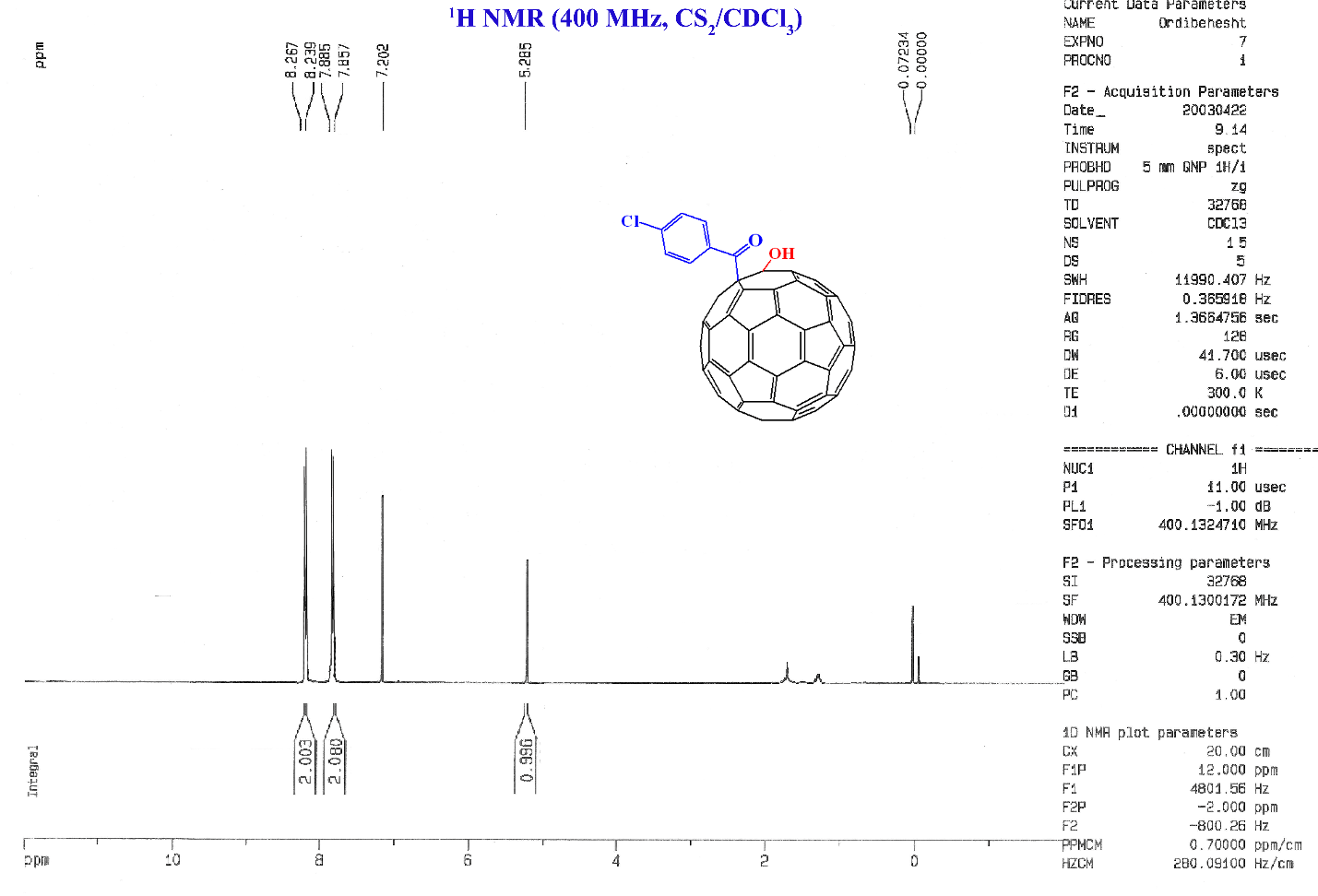


**Figure S1a**. ^1^H NMR of CB-C_60_


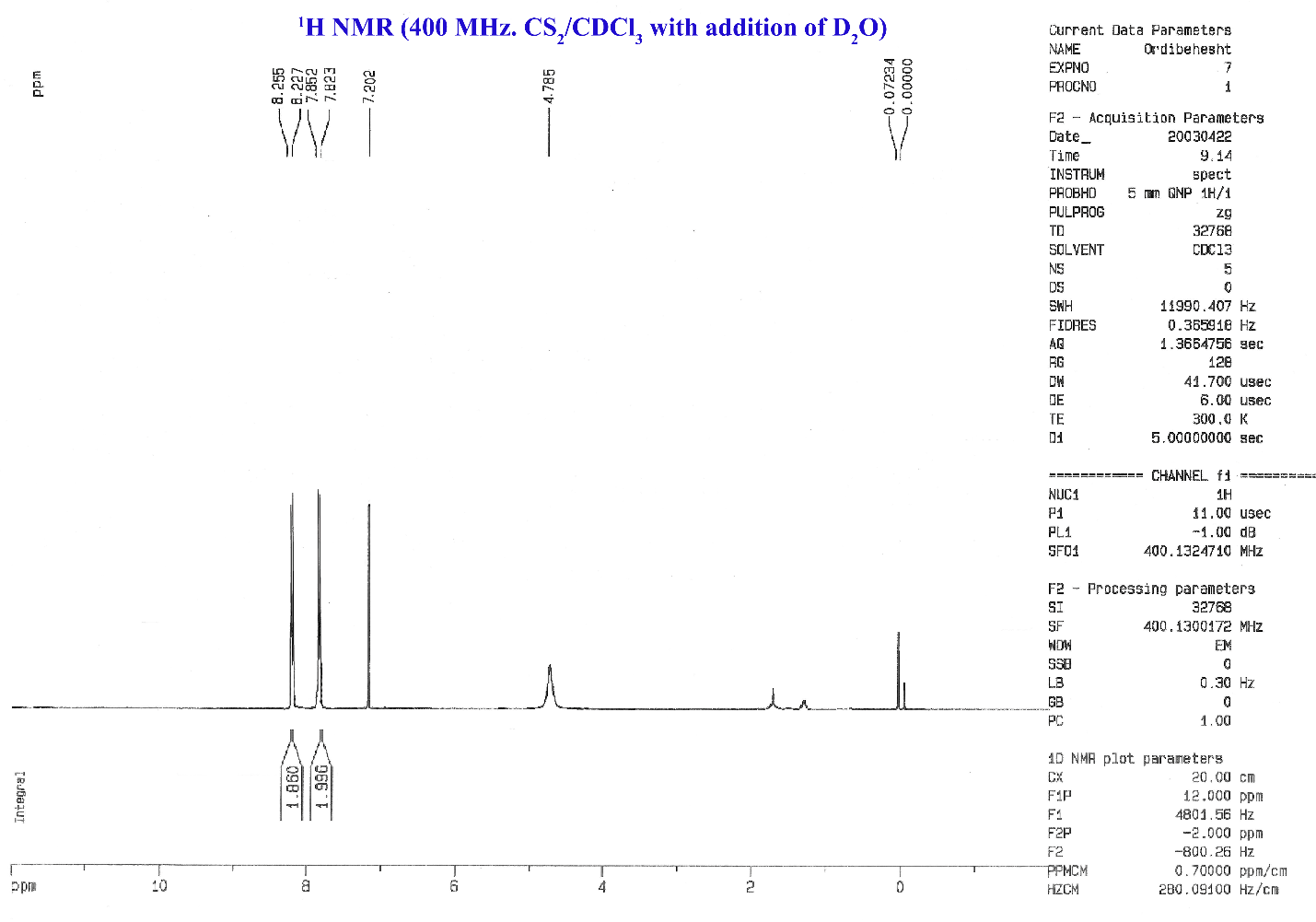


**Figure S1b**. ^1^H NMR of CB-C_60_ with addition of D_2_O


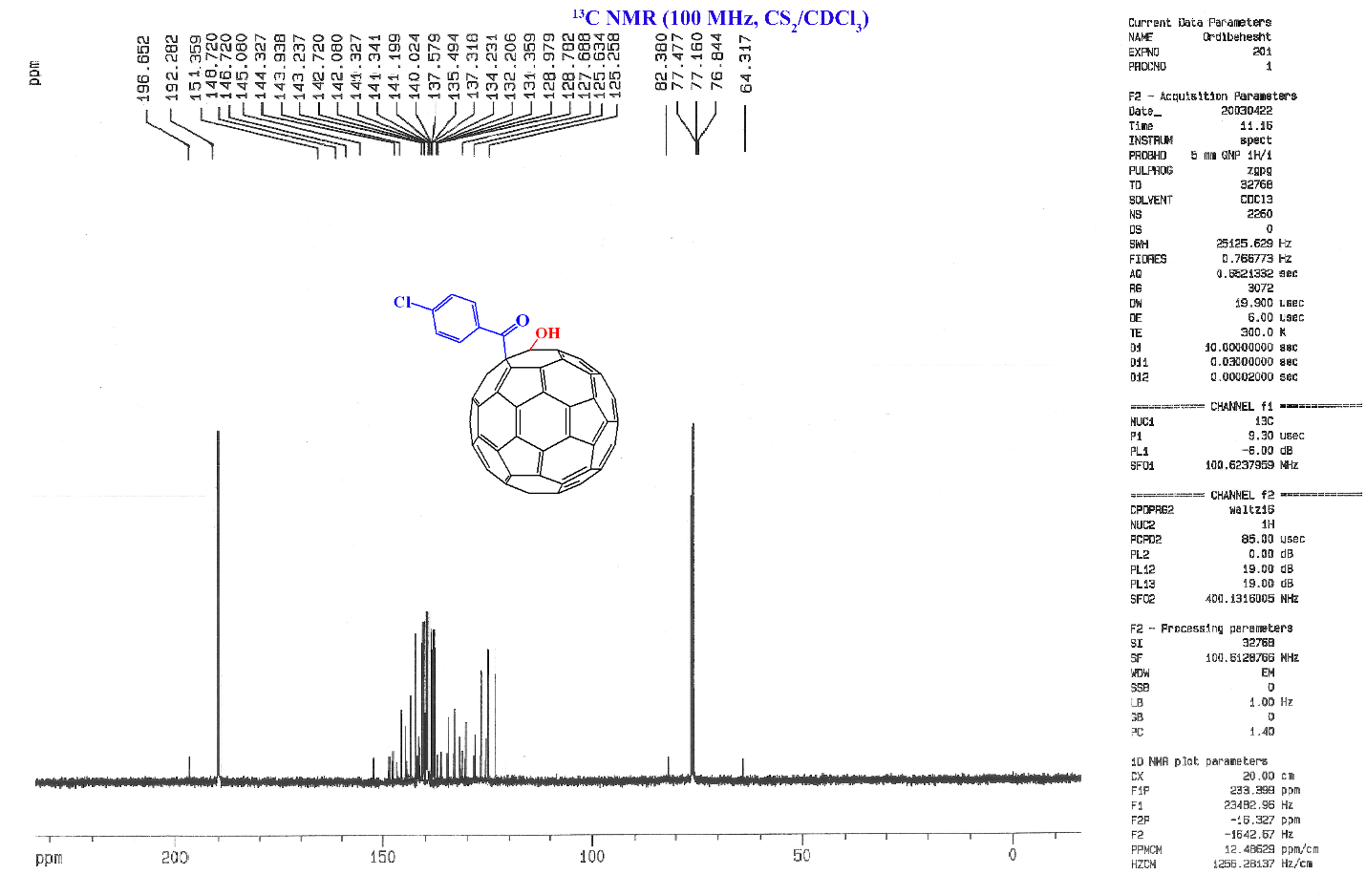


**Figure S1c**. ^13^C NMR of CB-C_60_

**Figure S1d**. FT-IR spectra of C_60_, CB, CB-C_60_

**Figure S2**. FT-IR spectra of C_60_, CB, CB-C_60_, MWCNTs, and C_60_-CNTs hybrid

**Figure S3**. XRD spectra of C_60_, CNTs, and C_60_-CNT mixture


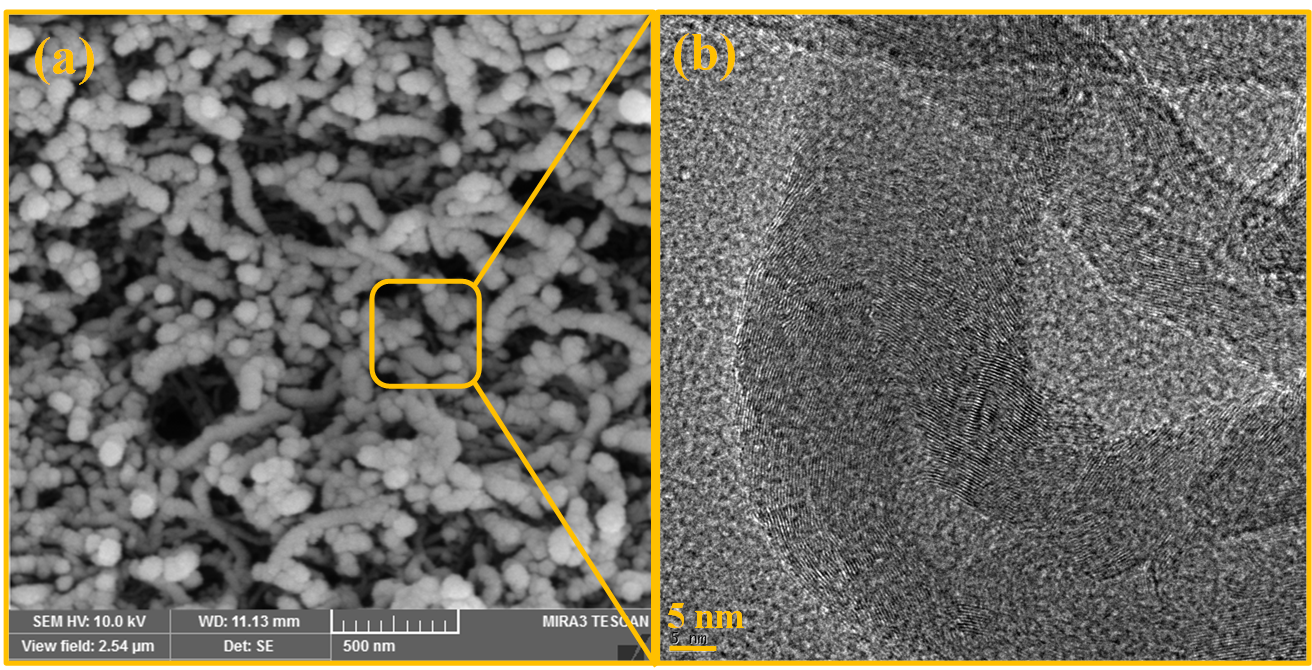


Figure S4. (a) SEM and (b) TEM images of CNTs


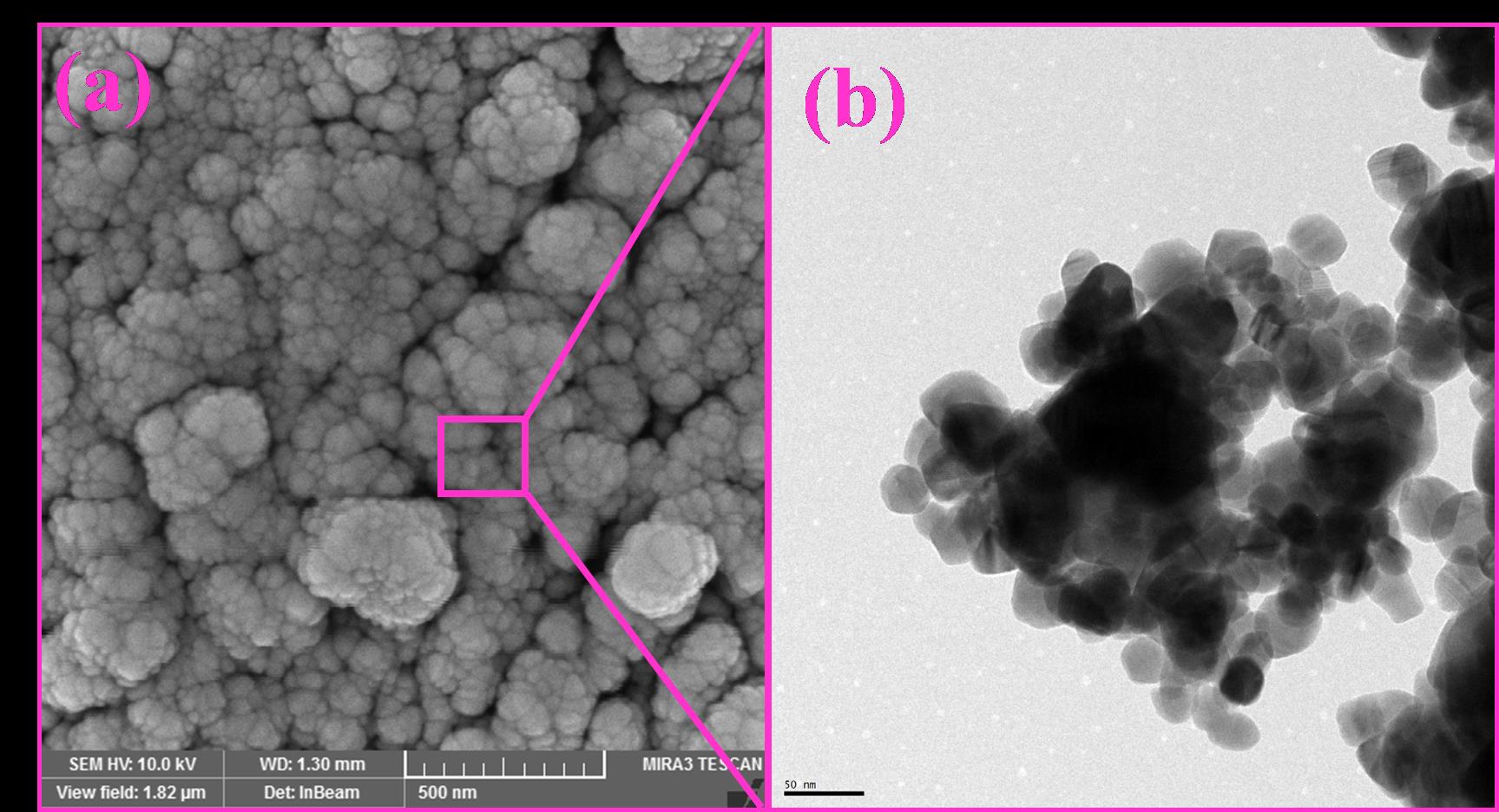


Figure S5. (a) SEM and (b) TEM images of CB-C_60_

**Figure S6.** the ECSA of C_60_-CNT hybrid, C_60_-CNT mixture, CNTs, and CB-C_60_ in 1 mmol L^−1^ [Fe(CN)_6_]^3−/4−^ and 1 mol L^−1^ KCl solution at scan rate of 10 mV s^−1^.

**Table S1.** Comparison of H_2_O_2_ production rate and current efficiency

| Catalysts | pH | H_2_O_2_ Production rate  (mgL^-1^h^-1^) | Efficiency  (%) | Refs |
| --- | --- | --- | --- | --- |
| Carbon fiber | 7 | 8.61 | 52 | [^1^](#_ENREF_1) |
| Anthraquinonemonosulphonate/polypyrrole | 6.3 | 25.1 | 55.5 | [^2^](#_ENREF_2) |
| Modified graphite felt | 7 | 87.9 | 80.8 | [^3^](#_ENREF_3) |
| N-doped graphene | 7 | 247.5 | - | [^4^](#_ENREF_4) |
| CNTs | 6 | 84 | 32 | [^5^](#_ENREF_5) |
| CNTs/carbon black | 7 | 1047.13 | - | [^6^](#_ENREF_6) |
| CNTs/graphite | 3 | 4.03 | - | [^7^](#_ENREF_7) |
| Modified carbon felt | 7 | 320 | 51 | [^8^](#_ENREF_8) |
| N-doped porous carbon | 1 | 3.3 | 65.2 | [^9^](#_ENREF_9) |
| Anthraquinone/  carbon black | 0.7 | 354 | 26.3 | [^10^](#_ENREF_10) |
| N-doped graphene | 7 | 450 | 75.2 | [^11^](#_ENREF_11) |
| Co/Carbon | 0 | 13.6 | 80.0 | [^12^](#_ENREF_12) |
|  | 7 | 1.45 | - |  |
| N-Doped graphene | 7 | 7.64 | 43.6 | [^13^](#_ENREF_13) |
| N-Doped Mesoporous Carbon | 7 | 7.82 | 55 | [^14^](#_ENREF_14) |
| NiFe@C/graphite | 3 | 5.68 | - | [^15^](#_ENREF_15) |
| Graphitic ordered mesoporous carbon | 13 | 21.92 | - | [^16^](#_ENREF_16) |
| HPC-H24 | 1 | 3027.4 | 80.9 | [^17^](#_ENREF_17) |
|  | 7 | 843.2 | 70.8 |  |
| C_60_-CNT hybrid | 3 | 4834.57 | 82.6 | this work |

**Reference**

1 Choi, J., Hwang, S. H., Jang, J. & Yoon, J. High yield hydrogen peroxide production in a solid polymer electrolyte electrolyzer with a carbon fiber coated mesh substrate. *Electrochem. Commun.* **30**, 95-98, (2013).

2 Zhang, G. *et al.* Oxidative degradation of azo dye by hydrogen peroxide electrogenerated in situ on anthraquinonemonosulphonate/polypyrrole composite cathode with heterogeneous CuO/γ-Al_2_O_3_ catalyst. *Appl. Catalysis B.,* **106**, 370-378, (2011).

3 Zhou, L., Zhou, M., Hu, Z., Bi, Z. & Serrano, K. G. Chemically modified graphite felt as an efficient cathode in electro-Fenton for p-nitrophenol degradation. *Electrochim. Acta* **140**, 376-383, (2014).

4 Yang, W., Zhou, M. & Liang, L. Highly efficient in-situ metal-free electrochemical advanced oxidation process using graphite felt modified with N-doped graphene. *Chem. Eng. J.* **338**, 700-708, (2018).

5 Gao, G., Zhang, Q., Hao, Z. & Vecitis, C. D. Carbon Nanotube Membrane Stack for Flow-through Sequential Regenerative Electro-Fenton. *Environ. Sci. Technol.* **49**, 2375-2383, (2015).

6 Yu, F., Chen, Y. & Ma, H. Ultrahigh yield of hydrogen peroxide and effective diclofenac degradation on a graphite felt cathode loaded with CNTs and carbon black: an electro-generation mechanism and a degradation pathway. *New J. Chem.* **42**, 4485-4494, (2018).

7 Khataee, A. R., Safarpour, M., Zarei, M. & Aber, S. Electrochemical generation of H_2_O_2_ using immobilized carbon nanotubes on graphite electrode fed with air: Investigation of operational parameters. *J. Electroanal. Chem.* **659**, 63-68, (2011).

8 Pérez, J. F. *et al.* Electrochemical jet-cell for the in-situ generation of hydrogen peroxide. *Electrochem. Commun.* **71**, 65-68, (2016).

9 Fellinger, T.-P., Hasché, F., Strasser, P. & Antonietti, M. Mesoporous Nitrogen-Doped Carbon for the Electrocatalytic Synthesis of Hydrogen Peroxide. *J. Am. Chem. Soc.* **134**, 4072-4075, (2012).

10 Valim, R. B. *et al.* Electrogeneration of hydrogen peroxide in gas diffusion electrodes modified with tert-butyl-anthraquinone on carbon black support. *Carbon* **61**, 236-244, (2013).

11 Su, P. *et al.* Electrochemical catalytic mechanism of N-doped graphene for enhanced H_2_O_2_ yield and in-situ degradation of organic pollutant. *Appl. Catalysis B.,* **254**, 583-595, (2019).

12 Barros, W. R. P., Reis, R. M., Rocha, R. S. & Lanza, M. R. V. Electrogeneration of hydrogen peroxide in acidic medium using gas diffusion electrodes modified with cobalt (II) phthalocyanine. *Electrochim. Acta* **104**, 12-18, (2013).

13 Han, L. *et al.* In-Plane Carbon Lattice-Defect Regulating Electrochemical Oxygen Reduction to Hydrogen Peroxide Production over Nitrogen-Doped Graphene. *ACS Catalysis* **9**, 1283-1288, (2019).

14 Sun, Y. *et al.* Efficient Electrochemical Hydrogen Peroxide Production from Molecular Oxygen on Nitrogen-Doped Mesoporous Carbon Catalysts. *ACS Catalysis* **8**, 2844-2856, (2018).

15 Sajjadi, S., Hasanzadeh, A. & Khataee, A. Two-electron oxygen reduction on NiFe alloy enclosed carbonic nanolayers derived from NiFe-metal-organic frameworks. *J. Electroanal. Chem.* **840**, 449-455, (2019).

16 Sa, Y. J., Kim, J. H. & Joo, S. H. Active Edge-Site-Rich Carbon Nanocatalysts with Enhanced Electron Transfer for Efficient Electrochemical Hydrogen Peroxide Production. *Angew. Chem. Int. Ed.* **58**, 1100-1105, (2019).

17 Liu, Y., Quan, X., Fan, X., Wang, H. & Chen, S. High-Yield Electrosynthesis of Hydrogen Peroxide from Oxygen Reduction by Hierarchically Porous Carbon. *Angew. Chem. Int. Ed.* **54**, 6837-6841, (2015).
